# Miscellanea Medica

**Published:** 1861-04

**Authors:** 


### Art. XIII.—MISCELLANEA MEDICA.

### I. DR. GILBERT SKEYNE, MEDICINAR TO JAMES VI. OF SCOTLAND.

The Bannatyne Club has recently published two highly interesting tracts, the authorship of one of which is due, and of the other is attributed, to a certain "Maister Gilbert Skeyne, Doctour in Medicine," who flourished in Scotland in the sixteenth century, He was the fifth son of one James Skeyne, "Notary and Farmer," who was killed at the battle of Pinkie-cleugh, and who was a descendant of "a small race of barons whose origin is lost in antiquity." One branch of the family is still nobly represented. Gilbert, born about the year 1522 or or 1523, received the customary education at the Grammar School and King's College, Aberdeen, and graduated as a Master of Arts; after which he applied himself to the study of medicine, took a doctor's degree, and in 1556 was appointed "Medicinar," or Professor of Medicine in King's College. While holding this position he published his tract on "the Peste," and this has now been reprinted by the Bannatyne Club, "page for page, from perhaps the only copy known, preserved in the Advocates' Library." He subsequently received other collegiate honours, but in 1575 he removed to Edinburgh. It is suggested that he was probably induced to take this step by his little tract having brought him into notice. He must have attained some celebrity in the practice of his profession, as on the 16th of June, 1581, he was appointed doctor of medicine (Medicinar) to His Majesty King James VI., and had granted to him an annual pension "of the sowme of twa hundreth pundis," money of the realm. In the same year, also, he was selected, together with Mr. Gilbert Moncrieff, surgeon, to inspect the Earl of March's person, in the celebrated divorce question between that nobleman and Dame Elizabeth Stewart, Lady Lovatt.

Doctor Skeyne appears to have retired from practice in 1593, and he died in 1599, leaving no family, but survived by his widow.

Of the two tracts now published, one entitled, *Ane Breif descriptioun of the qualities and effectis of the well of the woman hill besyde Aberdene*, is of local rather than general interest. The author's name is not attached to the tract, but the Secretary of the Bannatyne Club considers that it bears internal marks of being the production of Dr. Gilbert Skeyne. It bears the date of 1580 on the title-page, and it is the earliest known topographical tract connected with Scotland.

The tract on the Plague is of singular interest, not only from its containing an admirable summary of the opinions which may be supposed to have been entertained by Scottish physicians about the middle of the sixteenth century, on the nature and causes of the pestilence, but also from its being a very curious specimen of early popular medical literature.

The title-page of this tract reads thus:—*Ane Breve Descriptiovn of the Pest qhair in the causis, signis and sum speciall preseruatioun and cure thairof ar contenit. Set furth be Maister Gilbert Skeyne, Doctoure in Medicine. Imprentit at Edinburgh be Robert Lekprevik. Anno Do.* 1568. A succinct description of this little dissertation cannot fail, we think, to interest our readers. As many of them, however, might perchance stumble over some of the Scottish words and phrases, and might also have no dictionary of the language at hand to help them, we shall be pardoned, perhaps, for substituting in the quotations we shall give, English for Scottish words, although in so doing we sacrifice, in no small degree, the *crispness* (so to speak) of the writing.

Dr. Skeyne prefaces his tract with the following observations "to the Readar:"

"Since it has pleased the inscrutable Counsel and Justice of God (Benevolent reader) that this present plague and most detestable disease of Pest be lately entered in this Realme, it becomes every one in his own vocation to be not only most studious, by perfection of life, to mitigate apparently the juste wrathe of God toward us, in this miserable time: But also to be most courageous in suffering travail, for the advancement of the common wealth. I being moved in that part seeing the poor of Christ in lack, without assistance of support in body, all men detesting inspection, speech, or communication with them, thought it expedient to put shortly in writing (as it has pleased God to support my sober knowleage) what becomes every one both for preservation and cure of such disease wherein (good reader) thou shall neither abide great erudition nor eloquence, but ony the sentence and judgment of the most ancient writers in medicine expressed in vulgar language without polite or affectionate terms. And howbeit it become me rather (who has bestowed all my Youth in the Schools) to have written the same in Latin, yet undertanding such enterprises had been nothing profitable to the common and vulgar people, thought expedient and needful to express the same in such language as the unlearned may be as well satisfied as Masters of Clergy. Which being acceptable and allowed by the Magistrates of this Noble Burgh, conform to my good mind, shall, God willing, as occasion and time suffers, treat this same argument at most length, which presently for utility of the poor, and shortness of time, is moved to set forth almost rude and imperfect, not doubting, gentle Reader, but thou wilt approve the same with such like mind as the poor Woman's oblation was approved by the Good Lord, who may preserve thee in health of soul and body for ever and ever. So be it."

Following these remarks is, "Ane compendious descriptioun of the Pest," and, in the first Chapter, a Pest is described as "the corruption or infection of the Air, or a venemous quality and most hurtful vapour thereof, which has strength and wickedness above all natural putrefaction, and being contracted first most quietly infects the spiritual parts of man's body, thereafter the humours, putting soreest at the Natural Humidity of the heart which, suffering corruption and fever most wicked (pernicious), quietly and thief-like strikes the patient, whose body exteriorly appears well at ease, but interiorly is most heavily vexed." It is a feverish and most cruel infection, in sundry ways striking down many hastily. It is, therefore, a most vehement and high disease, and most dangerous, "because it is difficult to know all things which make a man propense to become Pestilential. Always, whatever has its cause from the Heavens, or corruption of Air, is properly, by most learned, called a Pest; and whatever is generated within us, or of other causes, is called a Malignant Fever."

Chapter II treats of "The Causis of Pest"—

"It were difficult and tedious," writes the author, "to treat of all the causes of a Pestilence. Therefore, at this present, I shall commemorate the principal only, by the which the rest may be understood.

"Certain it is, the first and principal cause may be called and is a scourge and punishment of the most just God, without whose disposition in all things other second causes work nothing. So the Heaven, which is the admirable instrument of God, blows that contagion upon the face of the Earth, as when the most noxious Starres to mankind convene, which by Astrologers are called unfortunate. Or when Comets, with other wicked impressions, are generated and preserved in the air, which, of itself, being most simple substance, and so incorruptible and necessary for man's life, natheless receives and admits, both from the Heavens and inferior Elements, many infections and corruptions which are the seed and chief causes of sundry diseases which are called epidemical, and these causes in most part are universal. Inferior causes are those which occupy a Realm, a people, a City, or a house thereof. Cause, therefore, is standing water, such as Ditch, Pool, or Loch most corrupt and filthy; Earth, dung, stinking Closets, dead Carrion unburied in especial of mankind, which, by similitude of nature, is most noxious to man, as every brute is most infecting and Pestilential to their own kind. Further continual showers of wet with great rushing wind, or the same blowing from pestiferous places. The cause of Pest in a private City is stinking corruption and filth, which occupies the common streets and ways, great smoke of coals without wind to despatch the same, corruption of Herbs, such as Kale and growing Trees, Moist heavy savour of Lynt, Hemp, and Leather steeped in Water. A private house, infected either of stinking closets or corrupt Carrion therein, or near by, or if the inhabitants have frequented other infected Rooms, or drinking corrupt Water, eating of Fruits or other meats which are corrupt, as we see daily the poor more subject to such calamity, nor the wealthy, who are constrained by poverty to eat evil and corrupt meats, and diseases contracted thereof are called Pandemial. In every one the cause is abundance of corruptible humours collected and generated of meats and drink which of any light cause become corrupt, in man's body as wicked as deadly poison. Finally and principally, infected Air, which all men draw of by inspiration of necessity for continuation of life. By the which, first the spiritual parts, secondly the humours and natural parts are sore put at, in some hastily, in others slowly or never, as one or another is accustomed to diversity of meats as the body is prepared and propense to corruption, and finally as dwelling and passion of the foresaid causes favours."

Chapter III treats of "The Signis of Pest." That is to say, the forerunners or indications of approaching Pest, and our author first observes that, "be cause the signis of the Pest to cum, pertenis to preseruatioun fra the same it becumes to treit thame at mair lenthe." The "first, truest, natural sign," he tells us, is continual wet without winds in the last part of Spring or beginning of Summer. Great and persistent heat, or Southerly Winds with turbid, misty air without wet, signify "a Pest to come in the Autumn next following." The "secund signe" is when Eclipses of the Sun are great and frequent, when Comets, or "fyrie inflammationnis," or falling stars, are seen, "for such things proceed and are generated of great drought, and hot fiery Vapours, which corrupt the Air, especially in the time of Autumne." If growing trees appear to wither (birne) it is a more certain sign of the calamity being at hand. "If the Air perseveres long time dry, as full of powder, with thik dry Clouds (as notably appeared all this last Summer) shows a Pest to follow of such nature." When the Air appears troubled and thick in Autumn and Winter as if wet were to follow, and wet then follows, that "constitution" is assuredly most corrupt. A pest in Summer is indicated when the Spring is dry and cold, southerly winds, with perturbed air, sometimes heat and other times cold, occurring afterwards, "which also signify Pocks and Measles and such like diseases of the body to follow."

"Both the former invade at all times of the Year, but least in Winter and Spring, ofter in Summer, oftest of all in Autumn, which most notably may be examined exposing fresh Bread to the Air one night, which if it corrupt most certainly the pest is at the door, if it be not already entered, as frequent mad Dogs prognosticates famine, which by infection of Air or Water become mad. In like manner Wolves entering in a town, with continual molestation, is sign of madness, for over great audacity shows phrensey, and by the same cause, that brutes, become furious or degenerate from their own custom of leaving, such humours corrupt in man's body as may generate a pest which are melancholy infected, by pestilential corruption of Air or Water. As before such times, the Sheep, which are more weak of nature nor man by death are afflicted, precedes also multitude of Frogs (Padokis) and Domestic Worms called in Latin *Blattæ*, which are generated of superfluous fat humidity, most repugnant to the health of man: as when the mole and serpent leave the Earth, being molested by the Vapour contained within the bowels of the same, which infection brings baith man and beast to death, the sooner if such increase of long time, and specially when the Domestical fowls become pestilential, it is a sign of most dangerous pest to follow, because when the drier and freer beast is infected, much more shall man who is more Humid of nature, and subject to less liberty, which may increase by baneful (wickit) mutation of the four times in the Year touching the principal qualities and natural constitution thereof, as a notable change of a natural day shall testify. Such like when pocks or such Pustules are frequent not only among bairns, but also among those who are of constant or declining age, great frequent south and south-west winds. If women with children through slight occasion part from their birth as when after vehement heat in Summer, wet follows, and abundance of Frogs appear, coloured gray on the back, of purple or any diverse colour on the wombe. As when Roses and Violets spring new in the Autumn, innumerable Worms, Flies, and serpents, great death of beast and fish, great dearth of Food, whereby men are constrained to eat evil and corrupt meats, most certain of all, hot and Humid constitution of the whole Year, the Sun at one hour shining, thereafter obscure with turbulent

Air, pronounce a Pest to follow. And therefore universal signs are to be observed."

Chapter IV. treats of "Quhat placis are maist Pestilential." Those places are more subject to pest, the writer asserts, which are near by the sea, "situate towards the south on height, whereby is abundance of corrupt Standing Water, where many dead are buried, where the ground is fat, and Evaporation increases most in time of Sun and Moon," and other celestial and planetary conjunctions. Those individuals are most subject to pest who have "abundance of thick corruptible humours," unless these humours be evacuated by bleeding, purgation, or the less sure preservative of a running sore. Such persons in particular are Children and Young Men and Women, "in their flower," who are of humid and hot temperament. Next are the hot and dry, last the dry and cold; "howbeit, the last are more difficult to cure than the first." No pest continues during more than three years, "either because it has not to urge or the Air being of most light substance may not suffer further putrefaction and which was corrupt before, further becomes not corrupt, as roasting once cannot be made raw again, and scarcely in so long time is the Air moved and renewed, and which was corrupt transferred in winds." Lastly, our merciful and omnipotent God puts measure to the pains of the wicked.

Chapter V. teaches "Quhariby corrupt be pest may be knawin;" and Chapter VI. makes known the "Signis of deth in pestilential persons." Chapter VII. treats largely of "Preseruation fra the Pest." We are instructed, as "the principal preservative care," first of all to supplicate God's grace and assistance through Christ; and secondly, "not pretermitting such support" as it may have pleased God to show us, reason "prescribes preservatives to consent in two things: first, to prepare the body apt to purgation; secondly, to make it which may offend weak in action or impression." The first object is to be obtained "by mundification and corroboration of the body," getting rid of all superfluous or corrupt humours by bleeding or purgation; the latter as being perfected many ways "by the intestines, urine, exercise, sweat, fasting, and difflation. The second object is to be sought by every one removing themselves from infected or suspected localities or atmospheres, and those—

"Who will not do the same, or moved by Christian Charity will not, must be studious to live in free Air, eschewing such constitution of Heaven and Elements as before is expressed to be most wicked, as cold at morning and evening, flavour of ditch or corrupt river, with all other filthy corruption, correcting the air universally or privately by fire and suffumigation made by aromatical materials, hot or cold, as the present constitution shall require, for certain it is, by experience of Medicinars observed at all times, that fire is an Antidote contrary the pest and all corruption."

A good list of aromatic herbs fit for suffumigation follows, with sundry complex recipes for combinations of the same, and their formation into "lozenges, thick powders, candles, or odorative paste (*pomis*)."

Other recommendations are, in summer :—

"Dwelling toward the northe, tempering the air in private lodgings, by aspersion of cold water mixed with vinegar, or clothes that be therein, and hung by the walls as tapestrie, leaves and flowers of cold herbs, which, by contrary qualities, temper and correct all pestilential corruption of the air, being used at the fairest hour of the day, opening door and windows towards the Septentrional parts: in other times of the year, towards the Orient, if nothing be repugnant thereto. Observing, also, that no domestic beast, such as Dog or Cat, stray abroad in time of pest."

Those in health who have to speak to suspected persons, and who refuse to take or who neglect other means, are directed to put in the mouth some anti-pestilential drug, as, for example, root of angelica, or to take a dose of some preservative preparation (several being duly prescribed) in the morning, &c. "But in such weighty disease, more profitable it were to use preservative remedies, in conformity to the logical cure before insinuated. . . ."

"Of exterior preservatives, fair clean clothes are most commendable, with oft changing thereof, and dwelling in lodging patent towards the occident or septentrion, far from corruption, wherein odorative trees, herbs, flowers, before expressed, are used in suffumigation, burning, or inspersion : no man passing forth from his lodging, until two hours after sunrise, noways in misty weather, without necessity compel, and that be after meat rather than fasting, anointing also the stomach, lever, and secret members with this ointment. Ric. olei rosati, omphacii unc. duas, olei de spica unc. semis, pulveris cinnamonis, gariophillorum, sing. drac. semis Rosarum sandalorum citronorum, cujusque drachma, cum modico cero et aceti sof. fiat unguentum molle. All meats preservative must be of good subtle substance, and dry in especial for those who are of a humid temperament. Travail and great fasting mundifies (I grant), but weakens therewith: as laborious exercise, or sweating, in corrupt Air are most dangerous; therefore, temperance, in travail or rest, sleeping or walking, meats or drink with temperate hilarity, are most commendable. Touching meats, flesh is most proper which generates laudable humours, and is of facile digestion, Such as Partridge, Pheasant, Laverock, Hen, Turtle-Dove, Kid, Mutton, *Cunning*, Veal, and suchlike others, using therewith Cloves, and powdered Cinnamon; all fish must be sodden with water, vinegar, powder of Cinnamon and Ginger. Abstaining from daily use of fat or sodden meats. Of herbs, the *Lettuce*, *Chichory*, *Purpie*, *Sourak*, *Pimpinell*, *Vetoun*, *Finkill*, *Anethe*, *Borage*, *Endive*, *Garlick*, in little quantity, *Raphorte* dissolved in Wine or Vinegar, may be used, preparing the same as becomes every one in their own nature. Of fruits, figs, bitter almonds, dry raisins, sour apple or pear, orange, citron, or lemon, capers, sour prunes or cherries, with daily use of vinegar or verjuice, with all sorts of meats, drinking clear, white, odorative wine, tempered with water, washing face, mouth, and hands, at morning, with wine tempered with rose-water, drawing at nose the decoction of the leaves of laurel, and anointing the ears with oil de spica, having in mouth the seed of citron, abstaining from sleep in daylight, Ire, crying, Venus plays, as from most dangerous enemies. Abstaining also from meats which corrupt hastily, as from variety of the same, which offend at all times, and especially fruits which be collected after contagious air, Swine flesh, Fowls that swim in water, using at morn a spoonful of the root of Aristoloche in powder with half wine, which resists to putrefaction and purges the heart pipes. Suchlike the powders of Unicorne, bol armenian, Harts horn, Pearl, Coral, Smaragde, Sapphire, Jasper, Ruby, drunk with convenint decoction, are most preservative."

Those who are constrained to visit the afflicted with plague must "first of all remove the opinion of death, but not the dread of God, therefore neither delight in peril, nor temerariously incur the same, without charity toward thy neighbour, or the glory of God (which is to be preferred to all thing) move thee."

Chapter VIII. treats of "Cure of the Pest;" and the tract concludes with an aspiration, that God would "indue us with the spirit of repentance, that unfeignedly we may convert us unto him, reforming our depraved and corrupt living in times past. And also apply ourselves in times coming, and the obedience of his Godly will and observing of his commandments, that thereby he may not only remove such punishment and Plague from us, but also that both rich and poor may live in such Godly and civil Society, as may be agreeable to his Godly will, that, finally, we may be participant of his kingdom, prepared for his Elect from the beginning."

### II. SPANISH MEDICAL PRACTICE IN THE SIXTEENTH CENTURY: ILLNESS OF THE SECOND WIFE OF PHILIP II.

We are indebted to Mr. Turnbull, late Calendarer of State Papers, for the following very curious letter, now for the first time published. It is from the English Ambassador in Spain to Queen Elizabeth, and the person to whom it relates was Elizabeth, daughter of Henry II. of France, and second wife of King Philip. The letter is remarkable, not only as exhibiting the state of medical science at that time in Spain, and as written by an envoy to a maiden sovereign, but as showing in the parts designed to be written in cipher, that, had her Majesty died, Philip designed to have married Mary Queen of Scots, a circumstance, we believe, that has never been noticed before :—

"*Sir Thomas Chaloner, Ambassador at Madrid, to Queen Elizabeth. Draft. Spanish Papers. State Paper Office.*

"Please it your Majesty. By letters of the 10th hereof, deposited in post by my servant Coldwell, amongst other things I gave the same advertisement of the Queen Catholic's sickness, which hath sithence proved such as I have thought it more than pertinent to signify the rest as followeth. On Saturday, at night, the 5th hereof, her Majesty having certain tokens that she was with child, reckoning from the 27th of May last, with great joy of the King and all folks here for that good adventure, insomuch as the Sunday following a solemn triumph was prepared to have showed her pastime, fell sick of a fever, at the first not much accounted of. But when it continued that daye, and all Sunday, on Monday the Spanish physicians (contrary to the opinion, as I hear, of her own doctor, an Italian) let her blood of the one arm, and the next day of the tother in great quantity, esteeming that the plucking away of her blood should allay the fervency of her ague, the event whereof proved contrary. For not only that letting of her blood (as should seem) exasperated her sickness, but what with the affliction thereof in these canicular days, or some other hidden cause, the second night after, her matrice opened, voiding a great quantity of that natural aliment which children in the mother's womb are nourished by, and the next morning after she dispersed of two female children, to the great sorrow of the King and all the Court here. With this her fever increased more fervent, with vomits, pains of the mother, and other very evil accidents, pronouncing no good hope; whereupon she was eftsoons the third time letten blood in the foot for allegement of the pain of the mother. For short, her sickness prevailing, notwithstanding all human remedies that these men could apply, at last about the eleventh day of her falling sick she began to rave and so fall into profound sleeps of lethargy, against the which neither the binding straight of her arms and legs, nor the searching to let her more blood at her nose and other places (for not one drop would follow, no more than of twenty ventoses or more applied all at once to sundry parts of her body), nor other inventions, could withstand the stronger violence of the burning fever and lethargy that held her. So far forth, as the fourteenth day, being the critical, the physicians pronounced she was not like (continuing those accidents) to escape. But yet for all this she a little amended, and came to herself again; so as on our Lady day, the 15th hereof, she was houselled, and the King (to comfort her the more) for company. And ever sithence with divers changes, one half of the day well, the other half evil, now giving hope almost assured of amendment, and now eftsoons relapsing, she passed Friday last the peremptory fourteenth; whereupon what joy the King and all folks took I need not write. But yet again, on Friday night her lethargy or profound sleep returned, with idle talk and other ill tokens. And, nevertheless, on Saturday morning she bettered again, but was in such wise all this while as that her ague more or less did leave her. So as towards the self evening she fell speechless, her mouth with an apoplexy drawn up to her ear, with the benumbing of her right arm and side, and in effect abandoned of the physicians, at this instant hour, *hora noctis undecima* 19 *Augusti*, she lieth at the mercy of God. The palace gates are shut; the lamentations in the Court, both of men and women, very tender and piteous; the chapel filled with noblemen, all praying upon their knees for her; and generally great and unfeigned moan made on all parts, as well for the favour her virtues and gentleness obtained at all men's hands, as for the good hope they had she should have been the mother of many princes, and lastly, for the fear men have lest as this matrimony allied two so mighty princes as her husband and brother be, so possibly this disgraced chance may breed I wot ne'er what hereafter, or at least accelerate it the sooner.

"This far furth I wrote the self Saturday night, having purposed in case the Queen had then deceased to have furthwith sent away this bearer with advice thereof to your Majesty. But on Sunday, towards morning, she began eftsoons to come to herself again, spoke, and took sustenance, and gave certain hope the worst was past. So as that Sunday, and also Monday last, account was made almost of her escape, but doubtfully, for even in the night her ague and other paroxysms more or less returned; so as what between now hope, now dread, with infinite processions of all sorts made here. At last, on Tuesday, the 22nd hereof, her paroxysms and other accidents, such as on Saturday afore, returned with like extremity, great number of ventoses applied prevailed not either to quicken her lethargy, which they term here *Modorro*, or else to draw blood from her. For brief, physicians and all others despaired of her health, but after Tuesday and Wednesday past with great danger and jeopardacy even to the even unction, at last, by means of a strong purgation of *Agaricum*, that made her have twenty-two stools, given at a venture in so desperate a case to purge those gross humours, she ever sithence has amended, and at this present is counted past danger, with no small rejoicement on all sides. And I for my part as glad as one *for avoiding of that casualty which my former letters of the* 11*th hereof do partly touch. Assuring your Majesty that amongst ambassadors and others in this Court, the account already was made the King would be a suitor that ways. But though this occasion be passed, I shall never well think the case well assured till that party be married out of these folks' reach, or of any other prince whose power may readily embrace any quarrel or claim that is pretended. Howbeit, in this behalf I believe the French would be as unwilling as we that these men should become our so near neighbours in Scotland, and would let it all they might, how ready soever those of Guise percase would be to set it furth.**

* The words underlined were intended to be written in cipher in the fair copy of the letter.

"On the latter part, a six days past here are certain news come of the Emperor Fernando's decease. The king a two days past in mourning apparel visited and condoled with his nephews here of Bohemia.

"Hitherto the King's gallies touching their intended enterprise are little spoken of; I wot not what it meaneth, but perchance for this year it may prove to nothing.

"In Corsica, Signor Pedro Corso so bestirreth himself as (the fortresses excepted) the whole island is at his devotion. If he hold out this winter, it is thought the next spring he shall have strong aid from the Turk.

"And thus, not wotting what more to write than I have written touching my humble suit for my return, driven to the extremity of all that may here be misliked and force a man to be parting hence, I pray God send your Majesty all glad and good things. From Madrid, the 27th day of August, 1564.

"T. Chaloner."

---

